# Virginia Medical Monthly

**Published:** 1874-12

**Authors:** 


*The Virginia Medical Monthly.* Edited by LANEON B. EDWARDS, M. D., Richmond, Va.

This is a new applicant for medical favor, which has been published since April last. The present number, (No. 7,) is the first which we have seen, and taking it as an example, the Journal seems eminently deserving of professional support.

